# Court-appointed expert consultation in Italy: an ethnographic study of parents’ beliefs, expectations, and experiences

**DOI:** 10.3389/fpsyg.2025.1668693

**Published:** 2025-10-15

**Authors:** Antonio Iudici, Francesca Rainieri, Tania Fiorini

**Affiliations:** ^1^Department of Philosophy, Education, Sociology and Applied Psychology of Padua (FISPPA), University of Padua, Padua, Italy; ^2^Institute of Psychology and Psychotherapy, Padova/Milano, Italy

**Keywords:** court-appointed expert consultation, party-appointed expert consultation, expertise, dispute, conflict, court, ethnopsychology

## Abstract

This study focuses on the process of Court-Appointed Expert Consultation (Consulenza Tecnica d’Ufficio, CTU) that parents involved in high-conflict separations must undergo. The CTU is an expert psychological assessment commissioned by the court to assist legal proceedings, aimed at providing judges with essential information for well-founded decisions. Given that this is a relatively new but rapidly growing field in Italy, also considering the increasing divorce rates, specific scientific literature on the subject is still limited. Therefore, the purpose of this research is to examine in greater depth how separated parents perceive and experience this process, considering the psychological aspects involved, their expectations, their evaluation of the investigation’s utility, and the motivations underlying their request. Through qualitative research based on semi-structured interviews, the results highlight that participants view the CTU as a useful decision-making tool for the judge, but also as a mediator and guardian of minors. However, parents often confuse the role of the CTU with that of a mediator. Many parents expect a “corrective” CTU for the other parent, based on the idea that the problem lies with the other party. Indeed, while the request primarily arises to protect the children, it is also aimed at countering the other parent. Parents described the process as a demanding but ultimately useful, offering opportunities for reflection and new insights. The CTU is ultimately seen as a “validation” of parental suitability and a tool for vindication, but also as not always resolving family conflicts. We believe these findings can be highly useful for the Courts that initiate the investigation, as well as for all professionals involved, including psychologists, lawyers, and juvenile judges.

## 1 Introduction

Numerous studies in the literature concur that parental separation and divorce are highly distressing transitions that destabilize the entire family unit, ranking among the most stressful life events for individuals and families (Pajardi et al., 2018; Bavagnoli, 2023; Deck et al., 2023). These experiences create critical psychological, emotional, and relational challenges for both parents and children (Zohoor and Kroll, 2008). A common difficulty is separating the couple’s relationship from their parental role (Giommi, 2002), resulting in disruption of the educational processes of the involved minors (Malagoli and Lubrano, 2016; Agostini et al., 2011; Radetzki et al., 2022).

We also know that separation experiences can sometimes create dynamics in which children are unduly involved (Bernet, 2015; Gottman, 2017), often through false accusations (Mercurio, 2021), sometimes assuming a consolatory function, support, and complicity with the suffering parent (Davidson et al., 2014; Dijkstra, 2017; O'Hara et al., 2019; Lange et al., 2022). In many cases, minors are subjected to explicit and implicit pressures, including economic ones (Visser et al., 2017; Lamela et al., 2016; Cavanna and Chiara, 2021). We know that in cases of high and persistent conflict, there are serious effects on the developmental path of minors (Harold and Sellers, 2018; Verrocchio et al., 2018; Pajardi et al., 2019) and on post-traumatic stress symptomatology (Camisasca et al., 2016).

To protect their children, some parents end up not explaining what is happening, creating situations of uncertainty for which children are not emotionally prepared. Conversely, other parents embrace the idea of involving their children and speaking clearly with them, more for ideological reasons than for protective needs, resulting in a failure to respect children’s processing times (Sarrazin and Cyr, 2007; Shumaker and Kelsey, 2020). In some countries, there is a tradition of developing a management plan for the involved minors’ paths before separation, while in the Italian context, this occurs only rarely, and decisions about children are often made during emotional conflict or when parents’ capacities are undermined by psychological distress (Henry et al., 2011; Roma et al., 2018; Treloar, 2019).

Several authors emphasize that negative effects can be exacerbated during judicial separation (Polak and Saini, 2019; McHale and Carter, 2019; Fabricius and Luecken, 2007). Many studies have focused on family and extrajudicial mediation processes (Giommi, 2002; Ellis, 2022) or on the difficulties inherent in difficult separations between spouses (Johnston, 1994; Mahrer et al., 2018; Van Dijk et al., 2020; Iudici and Corsi, 2017).

Despite such evidence, very few scientific studies (Verde and Passoni, 2009) have dealt with what occurs during the court-appointed expert consultation (CTU) activity, a procedure that can be activated by judicial activity in the Italian context when the dispute between parents involves minors and jeopardizes their health and protection. This procedure is initiated when the judge must decide on issues beyond ordinary knowledge, requiring specialized expertise (Franchi, 1973), thus requiring a sector expert. Presumably, this deficiency is linked to a fairly recent activity, which spread in the 1970s, with an interdisciplinary character involving both psychology and law.

Given the considerable developmental risk situations described above, there is a need to better explore the experience of those who must undertake this path and the related psychological factors involved. The research questions concern how parents perceive the CTU, specifically, what are the participants’ theories. What is the basis for the request, how are parents positioned regarding the CTU’s activities, and what expectations do parents have of the CTU? How do parents evaluate the process during the CTU (progress) and at the conclusion of the process?

The general aim is to collect data that can promote an improvement in the consultation activities of the various professionals involved and the judges, and naturally to positively impact the health of minors involved in parental disputes. This research thus intends to delve into the experience of parents who, following conflictual separation or divorce, face the Court-Appointed Expert Consultation process through the legal system and are assisted by a consulting psychologist. The objective is to detect how parents configure the court-appointed consultation activity, particularly to understand what expectations parents harbor, how the request to initiate a consultation occurs (when the request is from the parents and not the judge), and how the experience is evaluated by those who participated, whether as the requester (the one who initiates the request) or the resistant (the one who is involved by the other parent).

## 2 Legal consultation and evaluation in the international context: main orientations

Child custody evaluations differ across countries but share the common goal of safeguarding children’s well-being (Kelly, 2014). In the United States, the “Child Custody Evaluation” is conducted by forensic psychologists or specialized social workers, who examine the family situation through interviews, observations, and psychological tests (Bow and Quinnell, 2001). The process typically includes interviews with both parents, children, and other significant figures in the child’s life, as well as observations of parent–child interactions. Experts may also gather information from external sources such as teachers or doctors. In the United Kingdom, the “Child Arrangements Order” with “Welfare Report” is prepared by CAFCASS, an independent organization that assesses the interests of children in family proceedings (Masson, 2014). In Germany, the “Sachverständigengutachten” is an in-depth expert opinion conducted by psychologists or pedagogists, which examines family history and parenting skills (Salzgeber, 2015). In France, the “Expertise psychologique” or “Enquête sociale” focuses on the family environment and the emotional stability of parents (Neyrand, 2011). Australia uses the “Family Report” or “Child Custody Assessment,” known for its attention to child safety and risk assessment (Cashmore and Parkinson, 2009). In Canada, the “Custody and Access Assessment” emphasizes parents’ ability to collaborate in the child’s interest (Bala, 2004).

Prevalent methodological approaches include multimodal assessment, data triangulation, ecological assessment, focus on the best interest of the child, evaluation of parenting skills, and risk analysis (Gould and Martindale, 2007). However, these practices present some documented limitations. Expert subjectivity can influence assessments, despite the use of standardized tools. Studies have shown variability in recommendations between different evaluators for similar cases (Emery et al., 2005). Assessments often provide only a brief ‘snapshot’ that may fail to capture long-term family dynamics (Kelly and Ramsey, 2009). The process can be stressful for families and may even intensify existing conflicts (Johnston et al., 2009). Moreover, high costs can limit accessibility, raising equity issues (Bow and Quinnell, 2004). The predictive validity of these assessments has been questioned by research (Emery et al., 2005). Cultural biases may emerge in the tools and methodologies used (Rohrbaugh, 2008). Other limitations include the potential focus on conflict rather than cooperation, long completion times, difficulties in assessing very young children, and the lack of systematic follow-ups to evaluate the effectiveness of recommendations in the long term (Kelly, 2014; Gould and Martindale, 2007).

## 3 Court-appointed expert consultation (CTU) and party-appointed expert consultation (CTP) in civil proceedings in Italy

Expert consultation is a cognitive intervention within the legal process, structured only in highly conflictual cases where the spouses’ positions are irreconcilable. In separation proceedings, especially those with high conflict, the judge needs to avail themselves of the technical support of a CTU expert in the field, typically a psychologist, psychiatrist, or child neuropsychiatrist, to understand the situation of minor children and analyze parental competencies (Gennari and Tamanza, 2017).

Here, the intervention of the consulting psychologist falls into two macro areas: the evaluation of parental capacities and ability to act (Iudici et al., 2020a), and the investigation of family relationships and child custody situations (Sammicheli, 2019). The expert’s purpose is to provide specialized psychological knowledge that exceeds everyday understanding and serves as a foundation for resolving legal issues.

According to the fundamental right of defense, parties have the option to be assisted by a Party-Appointed Technical Consultant (CTP), whom they nominate and who is tasked with verifying that the expert operations are conducted correctly (Salvini et al., 2008).

Party-appointed consultants (CTPs) safeguard the interests of the parent they assist while also collaborating with the court-appointed expert to protect the child’s welfare and monitoring that the consultative and expert activities are carried out according to criteria recognized in the scientific community and generally not adverse to their party (Salvini et al., 2008). At the conclusion of the assignment, the CTU is required to provide written responses to the posed questions in the form of a report or Court-Appointed Expert Consultation, which is a psychological evaluation indicating the best modalities for child custody (Sammicheli, 2019).

According to the civil code, decisions are made in the best interest of minors, to ensure they do not experience additional psychological distress beyond what may potentially occur during separation, and to safeguard their growth and development (Salvini et al., 2008). The judgment subsequently issued by the judge is transformative, aiming to overcome the conflict and relational difficulties between parents and between parents and children.

## 4 Method

### 4.1 Knowledge background

Because few studies address the psychological aspects of the Court-Appointed Expert Consultation process, we aimed to explore how parents perceive the process and the role of the expert. To investigate the existential dimension of individuals, the research was conducted based on the premises of the interactionist perspective (Mead, 1934; Blumer, 1969; Salvini, 2004; Iudici et al., 2020a; Iudici et al., 2020a), which considers it important to explore the meanings that people attribute to their lived experiences. The fundamental concept introduced by these authors is that our action in the world is guided by the meaning we attribute to things, people, and events. This meaning arises within discourses, in which subjects actively participate (Romania, 2012).

### 4.2 Research method

This research employs qualitative methods of social research (Flick, 2009) to highlight the qualitative aspects of the investigated experience, interpret the meanings that subjects bring to their experiences, and valorize their words (Hennink et al., 2020).

A qualitative methodology was chosen to capture parents’ opinions, beliefs, and interpretations of their experiences during the CTU process. A semi-structured interview was used as the research method to analyze the discourses and accounts related to the participants’ lived experiences. Open-ended questions allow the person to express their opinions and recount their experiences freely, unconstrained by options (Jenn, 2006). Simultaneously, the semi-structured interview grants ample freedom to the researcher, allowing discussion of all themes, collection of necessary information, and exploration of the interviewee’s point of view (Cohen and Crabtree, 2008) (Table 1).

**Table 1:** Track interview.

How would you describe the role and functions of a Court-Appointed Expert Psychologist (CTU)? What expectations do/did you have regarding the Court-Appointed Expert Psychologist (CTU)? Describe the reasons that led you to request/accept the Court-Appointed Expert Consultation (CTU). Describe what you intend/intended to pursue through the Court-Appointed Expert Consultation (CTU). What expectations do/did you have regarding the progress of the Court-Appointed Expert Consultation (CTU) process? How would you describe the progress of the process?

### 4.3 Participants, recruitment and data collection

The study recruited 31 parents as participants, including 15 women and 16 men, aged between 34 and 60 years.

The inclusion criteria adopted to allow participation in the research were the presence of separation or divorce proceedings, the completion of a CTU process, and the presence of one or more minor children. Participants were at different stages of the CTU process when they joined the research: 15 were awaiting the judge’s final hearing (formally open general activity), while the other 16 had already received the judge’s decision (formally concluded activity).

Here we describe the procedure defined for conducting the research.

First step: Involvement of psychologists who are experts in forensic consultation.

*Expert recruitment was conducted by writing to* var*ious expert psychologists registered in the national register of court-appointed technical consultants and briefly describing the research objectives. Some of the individuals approached declined the invitation, while some psychologists accepted it. They were sent a specific presentation of the project with a request to ask parents for their availability to be contacted by researchers.*

Second step: Experts inform parents involved in a CTU about our research.

The experts asked all parents under their care for a CTU if they were interested in participating in the research. This *for a period of approximately six months, three months before and after the start of the research, if they were interested. The experts then offered parents the opportunity to contact the researchers.*

Third step:

*Interested parents contact the researchers, who arrange a meeting to carry out the research.*

The appointment was scheduled at the end of the CTU investigation *(for those who were undergoing counselling at that time) and immediately for those who had already completed the investigation.*

Fourth step:

The researchers carry out the investigation.

*The researchers then contacted the parents and verified their characteristics for inclusion in the research. All participants, before taking part in the qualitative interviews, were sent informed consent forms, along with instructions about the research procedures, the identity of the researchers, and received answers to their questions.*

*All interviews were conducted remotely via the Zoom platform and lasted between 40 and 60 min, taking place between November 2022 and September 2024. At the beginning of each interview, participants were reminded of the possibility to review and withdraw their consent for data use at any point during the research, as well as the option to not answer questions if they felt uncomfortable doing so.*

All names were replaced with randomly generated codes to ensure anonymity. *Since the court-appointed technical consultation procedure is an institutional practice, we believe there are no significant differences in the cities and different regions (Lombardy, Tuscany, and Veneto) where the psychologists come from.*

Other notes:

All participants were involved by the researchers, having no impact on the investigation objectives or on the experts evaluation objectives.

The research investigations aimed at capturing beliefs about the activation of the CTU, the request, the expectations, and the ongoing and overall experience.

The role of the CTU professionals was only to request the availability of parents to participate in the investigation, which was then conducted independently by the researchers. There was therefore no specific interest in the CTU professionals, who did not have access to the data. Of these, 21 participants formally requested the initiation of the CTU (“requesters”), 9 accepted it passively (“resistants”), and 1 participant did not provide this information.

The research was approved by the Ethics Committee of the University of Padua with number 4736 (Tables 2, 3).

**Table 2:** Participants information.

	Participant code	Gender	Age	Occupation	Number of children	Duration cohabiting or married	Applicant
1	P1	F	56	Architect	3	7 years	No
2	P2	F	51	Secretary	1	18 years	Sì
3	P3	M	41	Entrepreneur	3	14 years	No
4	P4	F	41	Factory worker	3	15 years	Sì
5	P5	M	43	ASPP	1	3 years	Sì
6	P6	F	38	Office worker	1	5 years	Sì
7	P7	M	34	Cook	1	5 years	Sì
8	P8	M	38	Works in logistics	2	7 years	No
9	P9	M	53	Company manager	1	16 years	No
10	P10	M	57	Pastry chef	2	10 years	No
11	P11	M	41	Manager	1	11 years	Sì
12	P12	M	43	Police officer	1	7 years	Sì
13	P13	F	34	Unemployed	2	7 years	Sì
14	P14	F	55	Secretary	2	20 years	Sì
15	P15	F	57	Real estate agent	2	18 years	Sì
16	P16	M	60	Psychiatrist	1	15 years	Sì
17	P17	M	52	Real estate agent	2	14 years	No
18	P18	F	55	Freelancer	1	18 years	Si
19	P19	M	39	Entrepreneur	1	10 years	No
20	P20	M	36	Freelancer	1	7 years	No
21	P21	F	48	Office Worker	2	12 years	Sì
22	P22	M	55	Teacher	2	14 years	No
23	P23	F	47	Office Worker	1	13 years	Sì
24	P24	M	43	Architect	3	8 years	No
25	P25	F	49	Secretary	3	9 years	Sì
26	P26	M	55	Entrepreneur	2	19 years	Sì
27	P27	M	52	Factory worker	2	15 years	No
28	P28	F	50	Health and safety officer	2	20 years	Sì
29	P29	F	39	Office worker	2	13 years	Sì
30	P30	F	37	Cook	1	12 years	Sì
31	P31	F	44	Teacher	1	14 years	No

**Table 3:** Age and gender distribution of the study participants.

	31–50 years	50–65 years	Tot	Average¹	Std. Deviation Cam (Dev. St. C.)¹	Std. Deviation Pop. (Dev. St. P.)¹	Std. error mean¹	*p*-value²
M	9	7	16	46,37	7.55	8.03	2.07	
%	56.25%	43.75%	100%					
F	9	6	15	46.73	8.29	7.29	1.95	
	60%	40%	100%					
Tot.	18	13	31	46.55	7.80	7.68	1.40	
	100%	100%	100%					
	100%	100%	100%					

¹Mean, standard deviation and standard errors refer to the age of the participants. ²*P*-value refers to the mean ages of men and women.

### 4.4 Data collection

All interviews were conducted remotely via the Zoom platform and lasted between 40 and 60 min, taking place between November 2022 and September 2024.

At the beginning of each interview, participants were reminded of the possibility to review and withdraw their consent for data use at any point during the research, as well as the option to not answer questions if they felt uncomfortable doing so.

Recruitment was conducted by writing to various psychologists registered in the national register of court-appointed technical consultants and briefly describing the research objectives. Some of the individuals approached declined the invitation, while some psychologists accepted it. They were sent a specific presentation of the project with a request to ask parents for their availability to be contacted by researchers. The researchers then contacted the parents and verified their characteristics for inclusion in the research. All participants, before taking part in the qualitative interviews, were sent informed consent forms, along with instructions about the research procedures, the identity of the researchers, and received answers to their questions. All names mentioned were replaced with codes composed of random letters to ensure the anonymity of participants’ data. Since the court-appointed technical consultation procedure is an institutional practice, we believe there are no significant differences in the cities and different regions (Lombardy, Tuscany, and Veneto) where the psychologists come from. This is also because the inclusion criteria concern objective aspects that are transversal to the context of belonging.

For their recruitment, agreements were made with three psychologists working as Court-Appointed Technical Consultants in Florence, Milan, and Padua, who authorized collaboration with the researchers and identified individuals with suitable characteristics for this research. The research was approved by the Ethics Committee of the University of Padua with number 4736.

### 4.5 Data analysis

Subsequently, the material was reviewed and analyzed using the Positioning Analysis methodology theorized by Davies and Harré (1990), with the aim of highlighting the modalities employed by participants to narrate their experience.

The concept of ‘positioning’ comes from the cognitive psychology of social action and explores the explicit and implicit reasoning patterns in people’s interactions (Harré et al., 2009). Positioning represents the fundamental way in which a self and identities are inserted into social interactions at practical, emotional, and epistemic levels. Harré and Van Langenhove (1992) describe self-positioning and other-positioning, arguing that both are implicated in the same act, as positions are complementary to each other. They are reflexive with respect to social actions, meaning actors are positioned by social acts and the meaning of social acts depends on how actors are positioned, what rights and duties they have (Table 4).

**Table 4:** Criteria for analyzing discourse position (Davies and Harré, 1990; Harré and van Langenhove, 1992).

Language used	It examines the metaphors, idioms and linguistic expressions used in discourse to understand how they influence positioning. Metaphors, rhetoric, linguistic acts (e.g. complaints), verb tenses, expressions used, interpretations and implicit and assumed meanings (the “unspoken”).
Narrative lines and positional acts	It analyzes how people construct and use stories to position themselves and others: Narratives, accounts, and ways of describing one's position regarding something. It studies the specific actions that people take to establish or modify social positions: Actions and activities implemented in responding to questions or addressing a particular issue
Social and cultural context	It considers how the broader context influences the positioning of individuals.
Moral/normative prescriptions	The rights and duties that each person assumes or undergoes implicitly or explicitly.
Macro-themes	The plots of the subjects' stories, within which the sub-themes are found, which would be the positions taken by the subjects.

### 4.6 Validation of scientific data

Credibility was obtained by specifying the researchers’ cognitive references, namely the epistemological and conceptual references described above. A second aspect concerned familiarity with the data, given that two of the researchers had previously dealt with legal psychology and were registered in the register of court-appointed technical consultants. Furthermore, the data were analyzed by all the researchers involved in the study, first defining the themes and positions individually, then there was a comparison aimed at resolving doubts and differences detected in understanding the text. To define significant aspects, the recursiveness of the text was also evaluated. Regarding the transferability of results, the data classification criteria were described and made explicit through the positioning analysis criteria, aimed at detecting the language used (metaphors, linguistic expressions, etc.), the discursive and prescriptive modalities of a moral order, the most emerging themes (see Table 3). This allowed for internal uniformity upstream and then allows for a generalized process of possible data transfer, this specific method, which falls within the methodologies of text analysis, allows the use of data reducing subjective interpretations of those who perform the analysis. Furthermore, this method is based on an accurate collection of the text, word for word, thus increasing accuracy. The positioning of the interviewed person can be deduced from the reported text. This is why in the results there was considerable use of the text and discourse reported by the participants. Finally, in our case, the investigation is very specific, concerning a very defined institutional practice, therefore in this case it is easier to make observations on the characteristics of the participants involved. The reliability of our work is given by the coherence between the epistemological assumptions and the definition of the protocol of knowledge questions, which were prepared trying to obtain not so much the contents but the discursive processes used by the interviewed people. Reliability is also given by the relationship between the results and the discussion, oriented to bring out the implications of the text rather than giving standardized explanations. The researcher’s reflexivity part consisted of constantly monitoring the application of the method used. For example, during the analysis, when it was noticed that the answers were not congruent with the purposes underlying the questions, we proceeded to discard the answer or to confront the psychologists involved in the first phase of the research. Regarding data saturation, it occurred for theoretical reasons. The analysis was concluded after 31 based on the following three criteria: the answers tended to repeat themselves, the identified themes contained a wide range of references, and the text was developed in order to obtain congruent data with respect to the defined objectives and based on how the relationship between the sub-dimensions clarified the general positioning of the participants. The repetitiveness of the text was monitored by noting when the text began to repeat itself. This process was implemented by all three researchers. The team consulted, evaluating when both the themes and the positions tended to become recursive. To try to disconfirm this sharing process, three further interviews were organized in which no further significant data emerged. In some cases, the linguistic expression was different at the lexical level but not at the positioning level. The specificity of the objectives and the monitoring of recursiveness allowed for a definitive sharing regarding the closure of the analysis.

## 5 Results and discussion

### 5.1 The configuration of the CTU

#### 5.1.1 CTU as a decision-making tool

Several participants use the words “useful” and “fundamental” to define the psychologist, recognizing that without their help, the judge could not make an appropriate decision. This perception aligns with findings from other studies that have shown the crucial importance of the forensic psychologist’s role in the judge’s decision-making process in child custody cases (Bow and Quinnell, 2001). These words are used to position the psychologist as an auxiliary figure to the judge, tasked with resolving a specific situation. Some consider them a helpful figure in understanding how to deal with children, while many others see them as a mediator between spouses or as a protector of children’s needs.

For example, one participant who had favorably accepted the CTU states: “It’s a very useful figure for the judge, helping them make truly delicate decisions. Without a psychologist, I think it would not be possible. It’s very useful for understanding relational dynamics” (P4). However, from these words, we cannot discern the real utility of the CTU psychologist for the parents. We do not understand if it can be useful for reflection, change, or improvement; rather, it provides more of a description of their functions.

#### 5.1.2 CTU as a mediation tool

A word used to define the CTU as a third figure who reconciles spouses would be “Family Mediator.” However, as highlighted by Kelly (2014), there is often confusion between the roles of family mediator and court-appointed technical consultant. Kelly emphasizes the importance of maintaining a clear distinction between these professional roles, underlining that family mediation and forensic psychological evaluation are distinct processes with different objectives.

For example, some participants describe its functions as: “Role of family mediator, studying and understanding possible resources for the family and children in relation to custody” (P19); “I understood that it helps to reconcile parents when minor children are involved. Fortunately, it protects and preserves their needs” (P16). Participants grasp the CTU’s function of protecting minor children, but they do not seem to have a clear understanding of the difference between mediator, therapist, and Court-Appointed Technical Consultant. On the other hand, this figure is rather emerging, and in the literature, there are many more studies regarding the family mediation process, which is different from that of the CTU (Turchi and Romanelli, 2019).

Moreover, the psychologist is described as a third figure who observes silently and then draws conclusions, above the parties. Indeed, they are defined as an “external eye,” indispensable for solving problems that otherwise could not be seen from within: “An external eye is needed to ‘judge’, to analyze problems that otherwise would have no solutions. When you are inside, it’s hard to solve on your own and see things, but if a third person tells you, everything changes” (P8).

Implicitly, the subjects seem to express the need to be helped by someone who sees the situation impartially and objectively, to understand how to behave and to demonstrate their reasons. It’s as if the psychologist is invested with many expectations and hopes because the couple alone cannot resolve the problematic situation.

#### 5.1.3 CTU as a functional tool for minors

One participant configures the role of CTU as a tool oriented towards protecting the minor and not towards themselves as a parent, stating: “The CTU has a fundamental role in helping the minor, understanding even deeper and unconscious issues, and guiding the minor to understand, to autonomously resolve all this, with the aim of resolving their discomfort, positively overcoming the problems” (P24).

This excerpt seems to imply that the psychologist’s intervention helps only the child and not the family unit. Thus, the focus is shifted to a third person, outside the couple. On one hand, it seems to be an advantage as the parent directs their attention to the child’s needs; on the other hand, it’s as if the intervention does not concern themselves, as if it wasn’t meant for reflection, but only for another person to do so.

While it’s true that international literature emphasizes the importance of primarily considering the well-being of minors in custody evaluations (Emery et al., 2005), it’s also true that such attention in the observed text seems to be practiced more in an ideological sense or in opposition to the parents’ health (Turchi et al., 2022).

In any case, participants manage to grasp the function of the CTU as one who protects and considers the needs of the children. For example, one subject states: “My children’s needs would have been forgotten if there had not been the CTU psychologist” (P17). The past perfect tense is used, in conditional terms, as if the participant were predicting what would have happened without the psychologist’s intervention. The interviewee positions the detection of need based on the presence of the CTU, believing that the psychologist is helpful, but not specifying how.

#### 5.1.4 CTU as a guarantee tool

To describe the functions of a CTU psychologist, several people use the verb “should,” positioning the psychologist in a normative and prescriptive way. Thus, participants describe the psychologist’s functions based on what they believe they should do, attributing a role to them. Therefore, the subjects in question also position themselves normatively, as those who had to be evaluated by the psychologist, particularly if they were suitable in the role of parents.

For example, one person responds in this way: “In my specific case, the psychologist had to evaluate my parental suitability. I had been presumably declared unfit for my role as a mother, and therefore I had to prove psychologically suitable to independently manage the children” (P3). This participant implies that the CTU was requested by the husband to ensure that she could be suitable to care for the children. Parental suitability was a recurring theme, with some parents describing the process as if they had to pass a test and receive a ‘stamp of approval’ from the psychologist, thanks to a guarantor who evaluates them.

In reference to this, international literature highlights how the guarantee tool is the fulcrum of evaluation practices in the legal field, despite various evolutions over time (Ackerman and Pritzl, 2011).

### 5.2 The configuration of the request

#### 5.2.1 Promoting the protection of one’s children

Most often, the request arises from the need to protect minor children, due to a “difficult situation” according to some participants. The main motivation, therefore, seems to be to protect the children and prevent them from further suffering, as one participant states: “I believe my daughter needs to free herself from a great burden within her” (P28). Consequently, several participants express the objective of protecting and prioritizing their children’s needs, for example: “I wanted our daughter’s voice to be heard in this whole matter” (P12).

What emerges from various accounts is that the CTU is established precisely to protect minor children, thus positioning it as a useful means to achieve an end. In this regard, one participant states: “The children would be forgotten if not for the CTU” (P4). Some participants express their fears and guilt towards their children: “I wanted to find a way to make my children feel better, as they are the ones who suffer the most” (P27); “It’s not fair for children to pay for their parents’ mistakes” (P6).

Indeed, they seem to realize the effects that conflict between parents can have on children, even in the long term. This awareness reflects the results of numerous studies that clearly show how prolonged parental conflict can have significant negative psychological effects on children (McIntosh, 2003; Amato, 2010; Grych and Fincham, 2001; Hetherington and Kelly, 2002), including psychopathological ones (Harold and Sellers, 2018).

Then there are those who “fight” for shared custody of the children, opposed by the other parent who would prefer sole custody: “I was asking for the daughter, while my wife disagreed. I wanted to spend at least more time with her” (P14); “I wanted equal rights for our son and I wanted to spend more time with him” (P18).

Both these excerpts are taken from speeches made by fathers who had to accept the CTU due to accusations from their wives and who are trying to recover their relationship with their children.

#### 5.2.2 To counter the behavior of the other parent

We can observe a passivizing mode of speaking from some participants, with the attribution of blame to the other spouse and implicit accusations, as in this case: “My husband only accepted because I insisted, for him there were no problems” (P26).

Another participant lists the shortcomings of the ex-spouse without arguing and excluding themselves from the discourse: “The father’s behavior, the absence, the lack of responsibility towards the daughters, the approach” (P11). Thus, the attention is completely shifted to the other person, losing sight of the family system.

Among the objectives of some participants, there persists a desire for revenge against the other spouse, so in this case we cannot speak of a real objective, but of a desire shifted towards another person, as follows: “But I wanted him to be unmasked and for all the things he had done, especially to our children, to come out. He really made life impossible for them” (P7). We are always within a passive process of delegation.

“I wanted an expert to evaluate our situation and above all to make my husband reason and evaluate him as a suitable parent to take care of our daughter” (P16); “I would like the father to fully perform his role” (P20).

Here, from a narrative point of view, we can notice a deresponsibilizing positioning from which emerges a content of complaints about the other person’s shortcomings. It’s as if the CTU was requested only due to the fault and responsibility of the other person, without considering that the process must be faced together.

Participants believe that only the other person should achieve the objectives, so we cannot consider them real objectives, but delegations. Also in this case, this data confirms some other studies on the negative value of an accusatory positioning towards the other parent (Emery, 2012).

#### 5.2.3 Passive and obligatory acceptance

In this case, one of the two spouses positions themselves (or is positioned) as the one who opposes the separation and consequently also the CTU. It often happens that this parent ensures that even the children do not accept the separation, practicing a position of opposition to the other parent. The result is that the children lose contact with one of the two parents, usually frequenting only the one they live with. In this regard, one participant states: “The request arose because basically my ex-husband never accepted the separation and moreover did not make my children accept the separation. I still do not see them or hear from them even though the CTU seemed to be going well” (P21).

A recurring theme was children being drawn into the conflict, creating a form of triangulation, also confirmed by several studies in the literature (Patrizi, 2012; Johnston et al., 2009). Some participants argue that the children are manipulated by one parent, who would tell them what to declare before the judge to strike at the other parent: “She turned them against me and wanted them to declare in the CTU that they did not want to stay with their father because he was violent. The children’s psychologists said they were happy with their father” (P7).

#### 5.2.4 To improve one’s parenting skills

Some participants declare they want to: “Communicate more functionally with the ex” (P19) thanks to the CTU; “Be present and adequate parents for the children, fulfill one’s duty” (P23). These responses can be read thinking that the CTU can be truly useful if the participants position themselves as they declare. The declaration occurs when the people in question are still at the beginning of the process and are expressing their expectations. There are those who want to “Avoid conflict thanks to the CTU, which helps to reflect” (P3), a theme that is also confirmed by some studies in the literature. In fact, according to Patrizi (2012), despite the difficulties and implications of CTU work, such a process can become a useful opportunity for spouses to reflect on the family situation and the interests of the children. Some participants declare to “See things with different eyes” (P30), which means that the CTU in some cases can help promote the idea of positive change, emphasizing the difference between a before and after. Some authors have highlighted the importance of increasing co-parenting skills in the post-separation period, bringing long-term benefits for children (Pruett and DiFonzo, 2014; Consegnati et al., 2018).

### 5.3 The configuration of expectations

#### 5.3.1 The expectation of a corrective CTU

Several participants described the psychologist as having a corrective role, almost like an educator entrusted with adjusting problematic behaviors: “I expect them to understand the dynamics and correct the attitudes that can hinder decisions to be made for the children and limit the conflict between exes for the well-being of the minor” (P15).

In any case, participants implicitly express a request for help (for themselves or for the children or for the other partner), such as: “I expect them to be able to help our daughters with their relationship with their father” (P31); “I expect to be able to find a balance with our daughters” (P22).

These excerpts of discourse come from people who have requested the CTU for what they define as “failures” of the other parent and therefore harbor different expectations towards the process, but shift their objective towards another person. This tendency to focus on the shortcomings of the other parent has been widely documented in international literature. It is indeed known that many parents in conflict often tend to project responsibilities onto the other partner, hindering effective collaboration for the well-being of the children (Johnston et al., 2009). In fact, some declare that the process could serve the other partner for reflection. Here we note the tendency of some participants to speak only of the other partner and not for themselves, as if the process had been undertaken for only one of the spouses and not for both.

The hope of many is that the other spouse will reflect thanks to the CTU; consequently, they often use passivizing expressions, attributing blame and responsibility to their partner. This implies that the subjects undertake the CTU not for the family “we,” but because the consulting psychologist intervenes on the other partner, delegating to them the resolution of the problematic situation.

This dynamic is in line with what is reported in the literature, namely the fact that spouses fail to remain united as parents, even if separated, and to collaborate for the children. They struggle to separate the couple relationship from the parental one and to still feel part of the family after separation (Cigoli and Scabini, 2014). This difficulty has also been highlighted by other authors, who emphasize the importance of helping parents maintain effective co-parenting despite separation (Emery, 2012). Moreover, it seems that participants do not grasp the precise function of the CTU psychologist: they are not meant to help or judge, but rather to explore situations that go beyond the judge’s knowledge and provide a sort of snapshot, thanks to which decisions can be made.

#### 5.3.2 Expecting to understand what other roles do not see

Many participants express the need and desire to be recognized by the CTU psychologist, to demonstrate who they are, perhaps because this was lacking within the couple: “They really look at the problems and protect people. They look at the facets in the couple and in the minor that judges and lawyers do not see” (P11).

Regarding linguistic acts, participants’ polemics and complaints towards the judicial system, lawyers, and Party-Appointed Technical Consultants recur. In this case, participants express their opinions, specifying that they are introducing the personal dimension. Participants contrast the figures of CTP and lawyers with that of CTU, declaring for example: “The lawyer thinks about the interests of the client and not about protecting the minor, so the psychologist was really needed. They grasp nuances that legal lawyers do not grasp., with all due respect. They really look at the psychological state of the minor above all. They really look at the problems and protect people. The lawyer protects at a legal level and not psychologically” (P9); “The CTPs accused each other and this is ridiculous. They teach you how to be another person, they tell you how to respond and how to behave. The psychologist should understand clear signals, which lawyers often do not understand” (P2).

The use of the verb “should” positions the psychologist normatively, as if the participants were imposing a task on them. In making these speeches, participants used comparison methods, paralleling the different figures and especially explicating the differences. Therefore, it is interesting to understand how parents see the various professional figures differently. In fact, some authors argue that a clear understanding of the different professional roles can lead parents towards a more effective and less conflictual separation process (Kelly, 2014).

#### 5.3.3 Expecting less than the help received

Some participants declare that they had no particular expectations regarding the figure of the psychologist or that they were not very aware of it before undertaking the process. In most cases, there is then a change in positioning: from the few initial expectations, participants declare that the process then proved to be very useful. In this regard, one subject declares: “At the beginning, I did not even know what it was about. In hindsight, I realize it was the best solution” (P29).

In this case, indeed, the linguistic expression “in hindsight” is used to indicate that a change occurred between before and after the CTU intervention. A theme that often emerged is that the man is the figure most penalized by the judge, while the CTU, instead, seems to render justice to fathers. An emblematic phrase is this: “The mother, by default, always has all the rights and the father does not. It rendered me justice. It’s the best process” (P23).

This theme is also confirmed by the literature, which maintains that the mother has always been the figure to whom children are predominantly entrusted. Especially before the 2006 reform, when the criterion of exclusive custody was in force, the custody of minor children was the responsibility of the mother, penalizing the paternal figure (Patrizi, 2012). From 2006 onwards, with the reform of shared custody, the father has also been able to acquire importance as a caregiver (Gennari et al., 2016). This evolution of the paternal role in the context of custody has been widely discussed in international literature as well (Warshak, 2014; Fabricius et al., 2012; Pruett and DiFonzo, 2014). Several authors have highlighted the growing importance of paternal involvement and how this has been progressively recognized in custody processes in many Western countries (Lamb, 2010; Adamsons and Johnson, 2013; Nielsen, 2018). Probably, however, from the words of some participants, it is understood that within the judicial system there are still cultural legacies that lead to penalizing the paternal figure.

### 5.4 The evaluation of the court-appointed technical consultation (CTU) process: the CTU path

#### 5.4.1 Challenging but more useful than expected

Participants often described the CTU process as ‘challenging’: “At first it seemed like a useless and somewhat challenging process, but then it helped me understand the deep motivations that had pushed me to make this choice” (P17). However, we see that from an initial difficulty, the participant changes their positioning regarding the process, declaring that it proved useful, using the adverb “Instead.” Thus, their expectations were positively disappointed.

“I realize that I initially experienced the CTU as an injustice, but gradually I noticed that it was useful to understand that I needed to consider some things better, like my son’s need to be at peace” (P13).

What initially felt like an ‘injustice’ often transformed into an opportunity to better understand one’s child. And further, “I did not like the idea of having to air my private affairs, even though in the end I saw that it was useful because I was helped to grasp aspects that I usually do not consider” (P7).

This perception of initial fatigue, followed by a recognition of the usefulness of the process, is in line with what has been observed by other authors, who confirm its positive meaning (Bow and Quinnell, 2001).

The difficulty is not only experienced towards the process, but also towards one’s spouse, who seems to hinder it: “Very challenging because I had to defend myself based on nothing. It was useful for me and my children, not for the mother. The mother did not take advantage of it and did not understand the meaning of the process, she only used it for money. The civil relationship, as the judge wanted, does not exist” (P19). In this case, it’s interesting to note that the interviewee had not requested the CTU, but had accepted it, after being accused of physical violence by his ex-wife. Despite this, it seems that the CTU served him more than his partner, especially because from his words we can understand that the relationships remained conflictual even after the process. In several excerpts, despite the lack of collaboration between the two spouses and the consequent difficulty in facing the process, the interlocutors report having nevertheless taken the opportunity to reflect individually, and not as a couple.

Among the responses, one parent used the adjective “protective” to describe the intent to preserve the interests of minors: “As far as I’m concerned, I’m very happy because in a sense it does me justice. It’s protecting the child. At the moment I only thought about her and we are going towards the path I wanted and that she wanted too, that is, to be together more” (P13). This father recounts having taken the CTU to defend himself, to be able to spend more time with his daughter, which the mother seemed to prevent. The process seems to have served him and his daughter, while the other parent is not even mentioned in the discourse, as if they were not part of it.

“The CTU played a role as an opportunity for redemption to see one’s role appreciated: ‘The CTU was fundamental in having my right to be a father and be with my son recognized. Without this CTU, the mother would have continued to be the only one who could make decisions’” (P11).

Many then reiterate the fact that the CTU was undertaken only thinking of the children and their protection, as in this case: “It was unpleasant because I was annoyed to see my ex-partner and remember particularly arid years humanly. If E. had not been in childhood, I would have left my partner many years ago. I did not do it because I was aware that I would have lost my son, that she would have taken him away. No judge would have given a two-year-old child to a father” (P1).

In some cases, the consultation was requested to protect the children from serious dysfunctional behaviors of the other parent: ‘Reluctantly, I requested the CTU because it had become impossible to help my son defend himself against his father’s bullying’ (P24) and also ‘Without the CTU, it would never have come out that the mother needed help and needed to take medication. When I said it, I wasn’t listened to’ (P16).

#### 5.4.2 CTU as an opportunity to discover new aspects of oneself

Some participants argue that the CTU allowed for the emergence of content that might otherwise not have emerged. “It was very fundamental for me because in the end things came out that sometimes one does not even imagine thinking about. It brought out my character better” (P14). In this case, we can grasp the usefulness of the CTU as it allowed for self-reflection and better self-discovery, although the process did not then give the desired results. In fact, this participant then recounts: “I’m only sorry that it did not serve in the end. It had started to serve from the moment the CTU was interrupted” (P9).

The following texts account for how the consultation offered parents the opportunity to experience and recognize competencies in themselves that they did not believe they had: ‘I did not think I would be able to handle all the stress that the CTU required’ (P21) and also ‘There were many moments when I wanted to get up and leave, but I always managed not to do it’ (P4).

This ability of the CTU to facilitate new understandings has also been found by other authors, who emphasize how the evaluation process can often lead to significant insights for both parents and professionals involved (Bow and Quinnell, 2004; Gould and Martindale, 2007; Stahl, 2011; Austin and Drozd, 2012).

### 5.5 The evaluation of the court-appointed technical consultation (CTU) process: outcomes of the CTU process

#### 5.5.1 The CTU as a “validation” of suitability

The parents experienced the court-appointed expert assessment as a process aimed at validating their parenting skills.

“At the end of this process, the psychologist deemed me suitable for parental responsibility and produced a report highlighting the determining factors for the decision made. The CTU served to definitively decide the sentence, especially regarding the placement of the children. In fact, from there we decided how to manage them and how often they would see their father” (P12).

This is also and especially true for fathers: ‘Thank goodness the CTU recognized that I can be a dad. Without it, I always had to ask for the mother’s opinion. Now I can finally decide on my own whether to take my daughter to the swimming pool and whether or not to put a sweater on her’” (P29).

This participant responds using a descriptive style and recounts the usefulness of the CTU process as if referring to obtaining a stamp after passing a test. We must take into account that this participant had undertaken the CTU in 2004, before the enactment of the Shared Custody Law, at her husband’s request.

This perception of the CTU as a kind of ‘parenting test’ is also discussed in the literature, which warns against reducing custody evaluations to simple assessments of parental and personal suitability (Emery et al., 2005).

CTU as a tool for revenge and the assertion of certain rights brings out significant emotional aspects, also presented in terms of catharsis and personal redemption, infact someone said: “It served a lot, but today Italian justice is behind. The custody is joint but then it’s 5 days with the mother and 2 with the father, so I see little of the children. Money is used to get revenge on the other person. The children’s needs have been completely forgotten, except in the CTU. The children have been tossed around in important years for their growth. When they grow up, they will understand that no one did their good, except the CTU. The CTU exposed the mother, who was manipulating the children.” (P23).

“Everyone told me I was exaggerating, but the court-appointed expert proved that I was right to worry about my children. I will always be grateful for this because now I have even more confidence in my own assessments.” (P19).

Here returns the criticism of the legal system and, on the contrary, the idea that the CTU served for many reasons. When the interviewee says that no one helped the children, he refers to both the legal system and the mother, using an accusatory tone, as can also be seen from the verb “Expose.” We always notice this desire for revenge on the part of the spouses, who take the CTU for this purpose.

This dynamic has been explored by Bow and Quinnell (2001), who note how custody evaluations can sometimes be perceived by parents as an opportunity to “win” against the other parent. In line with the theme of fathers’ revenge, there are several interesting excerpts to report, such as: “The CTU helped a lot because without it no judge would have given me E. It’s a really powerful investigative tool, if done for the right period of time (not too short). It’s a very powerful tool for emotional investigation, on relationship mechanisms and individual parents’ abilities. It can overturn the now certain defeat on the part of fathers” (P21). This excerpt illustrates the strong emotional involvement of participants, who often described the consultation as decisive and life-changing.

#### 5.5.2 The CTU as a non-resolutive tool

However, some participants felt the process failed to produce the desired outcomes, such as: “There were not many, it seemed there was an improvement, but then in reality there was nothing. The custody of the children is 50%, only they are very angry about how the father is making them live it and they do not want to see me. They have been very ‘tampered with’ by people behind, including my ex-husband, who do not make them accept the thing. After 3 years we are still at square one” (P15).

Here too, it seems that the process did not go well due to the fault of one of the two spouses, who somehow prevents the children from seeing the other parent, rather than legitimizing them. The theme of parental alienation is often implicated in this type of situation and also requires a clinical analysis capable of bringing out the complex dynamics that can lead children to refuse contact with a parent after separation (Fidler and Bala, 2010). In fact, the frustration expressed regarding the definition of “conflictual couple” without an in-depth analysis of the conflict dynamics reflects some of the criticisms made of evaluations such as office consultation, which sometimes requires a detailed analysis of conflict dynamics, rather than resolutive evaluations (Johnston et al., 2009; Colacicco, 2018).

Another example of dissatisfaction with the outcomes is represented by the definition of “conflictual couple” that does not address the dynamics of the conflict: “It was said that we are a conflictual couple, but it was not considered that verbal and non-verbal aggressions always start from the mother: it’s impossible to talk to her, she yells at you... but we are conflictual” (P14). Here it seems that the father’s dissatisfaction is linked to a sort of equidistance of the CTU about the dynamics of the conflict, which he considers improper, as he feels he is suffering aggression rather than acting in an equal role with his ex-partner. When mothers complain, the mode is the same: ‘It wasn’t taken into consideration at all that the father is a violent person who raises his voice and more as soon as he is contradicted. I do not agree at all that they defined us as conflictual’ (P25).

“Sometimes the dissatisfaction is linked to expectations that are not in line with the objectives of a CTU, such as when parents complain about inadequate alimony amounts: ‘How can one be satisfied if I have to leave half of my salary to my ex’ and also ‘The CTU did not take into account that the father works a lot under the table and spends a lot on his vanities while leaving his son to suffer for the new backpack that I cannot buy him’” (P17).

The perception of the CTU as a not always resolutive tool reflects some of the challenges discussed by several authors. Stahl and Simon (2013) discusses how, despite the best intentions, custody evaluation processes may sometimes not completely resolve family conflicts.

## 6 Conclusion

This research aims to provide a starting point for reflection on a topic still underrepresented in literature and seeks to assist professionals working in this field, such as Social Services operators, for whom it could be useful to understand how to better prepare parents facing a Court-Appointed Technical Consultation (CTU) process.

Parents’ view of the CTU as ‘demanding but useful’ suggests that better preparation could substantially reduce the stress of the process. This could lead to a more positive and constructive experience for all parties involved, also in reference to the limitations of this type of assessment reported at the international level.

This need to improve CTU practice is supported by studies such as Bow et al. (2011), which highlighted the importance of continuous and specialized training for professionals involved in consultation and evaluation in the legal field.

For psychological consultants as well, starting from parents’ expectations and experiences, it could be useful to understand which themes to focus on more and how to approach them. If legislators and psychologists are aware of the images that parents attribute to the CTU process and the consulting psychologist, they can make various reflections on the underlying motivations and understand how to better approach the work. This could also be an opportunity to further align the legal and psychological sectors, which sometimes, according to some participants, do not converge.

It would be ideal for sector operators to clarify the different forms of intervention available to separated parents: technical consultation, family mediation, and psychotherapy.

This clarification could help parents better understand the process and manage their expectations more realistically, which is another limitation present in international practices.

This need for clarity has also been emphasized by Kelly (2014), who highlighted the importance of clearly distinguishing between different professional roles in the context of child custody disputes, benefiting users, involved professionals, and involved institutions.

Indeed, it is common during interviews for participants to express therapeutic requests and expectations of in-depth and prolonged parenting support, which are not the proper aims of a technical consultation. As a result, parents often misunderstand the process and enter it with incorrect expectations. It is not uncommon during consultation operations for one or both parents to ask the CTU to maintain professional secrecy on what is reported, demonstrating the role confusion acted out, since the CTU, being a public official, cannot guarantee secrecy on what is told to them, but on the contrary has the obligation to report the contents of the consultation to the Judge.

At the same time, given the requests and needs of the participants, it could be useful for a CTU psychologist to ensure that the Court-Appointed Technical Consultation also incorporates or integrates moments of mediation or clinical psychology (Iudici et al., 2019; Iudici et al., 2017), for example by involving other professionals in problem management from the outset. One could indeed consider an intersection between these similar paths, precisely to avoid delegating the protection of one’s personal and parental situation to the judge’s response.

It may be valuable—even innovative—to integrate elements of mediation or psychotherapy into the CTU process, potentially enhancing its overall effectiveness. This idea of an integrated approach between legal and clinical fields is supported by studies such as Pruett et al. (2012), which demonstrated the effectiveness of multidisciplinary interventions in the context of child custody disputes.

In this regard, the results of this research offer further insights to improve some of the limitations previously identified in parental evaluation practices present in international procedures. Firstly, the importance of a multimodal approach emerges, along with attention to the participant’s text and discourse, and the related triangulation of data, which could significantly contribute to reducing the subjectivity of the individual expert, one of the main limitations previously highlighted.

In this sense, the richness of comments during the interview, especially referring to the other person, suggests that various issues are still unresolved between ex-spouses, even years after separation. This data could help sector operators understand the best time to start a CTU process, to avoid dwelling too much on the conflict between the two spouses, rather than on issues related to the children. Furthermore, a task of the consulting psychologist is to consider the opportunity for further psychological and/or social assistance following the CTU, to monitor custody conditions and decisions made during the process (Gennari et al., 2016) without mistake (Iudici et al., 2015).

The idea of post-CTU monitoring, which emerged from the study, could increase the long-term effectiveness of the decisions made, thus improving the predictive validity of the assessments, another critical point previously highlighted in international practices.

In this regard, particularly interesting is the consideration (shared by several participants) that the CTU should have a longer duration. This would indeed facilitate the consolidation of that change which otherwise risks being interrupted along with the CTU. This temporal extension could provide a more complete and less ‘snapshot’ view of the family situation, thus overcoming one of the temporal limitations previously mentioned at the level of international practice.

This idea aligns with the recommendations of Stahl and Simon (2013), who emphasized the importance of in-depth and prolonged evaluations over time to fully understand the complexity of family dynamics.

In this regard, the practice of some Courts to follow the CTU strictu sensu with a monitoring period is often viewed favorably by parents, as monitoring allows maintaining the drive to act in relational modalities more in line with the objective of the children’s interest. Parents often report that once the procedure ends, if the CTU has not reconciled the parents’ positions, conflict quickly resurfaces.

Finally, the results highlight the importance of strengthening parenting skills, not merely resolving conflict. This shift in focus could lead to more lasting and beneficial results for the minors involved.

The results, although representative of the population requesting this type of evaluation, require further investigation as this research is also exploratory.

It is also necessary to consider various limitations of the present research, including the fact that a limited number of participants were recruited, due to the difficulty of involving them and accessing their stories. In the future, it would be interesting to replicate the study attempting to expand the number of people, so as to cross-reference data with as many experiences as possible, perhaps also from different regions of Italy to see similarities and differences. Moreover, it would be interesting to select participants who have undertaken the process with different CTU psychologists, with different training, so as to have more information in this regard. In this sense, our data are limiting since most people evaluated the CTU process in an overall positive way, having found competent and attentive psychological professionals who left participants with a good memory on a human level, and not just in terms of results obtained.

In this regard, future research could try to collect more data relating to the CTU psychologists in question, trying to understand their training, professional experiences, and studies, to understand if this affects the process and participant satisfaction. Another limitation of this research is the fact that participants were at different stages of the CTU process at the time of the interview, some were awaiting judgment, while others had already received it. Regarding the last research question, which aims to assess the overall experience of the consultation process with the psychologist, we believe that those who had not received a response from the judge had less time to reflect on the entire experience. Therefore, we believe that for these individuals, the results should be interpreted with caution.

Then in the future, it would be ideal to be able to interview both members of the couples to have both versions of the story. Indeed, in the present research, it was not possible to interview both spouses of each couple, thus losing the vision of the situation as a whole. A longitudinal design could assess whether changes following the CTU are sustained over time or only short-lived. Finally, further research on the topic could help professionals working in this field to understand how to make parents more aware of the process they will face, how to promote collaboration, and ensure that the CTU provides lasting results for all family members and promotes the health of the involved minors. The findings offer practical insights for improving CTU practice, making it more effective, equitable, and focused on children’s well-being. Implementing these recommendations could significantly improve the management of child custody in high-conflict separations.

## Data Availability

The original contributions presented in the study are included in the article/supplementary material, further inquiries can be directed to the corresponding author/s.
